# Confinement-Free Wide-Field Ratiometric Tracking of Single Fluorescent Molecules

**DOI:** 10.1016/j.bpj.2019.10.033

**Published:** 2019-10-31

**Authors:** Barak Gilboa, Bo Jing, Tao J. Cui, Maabur Sow, Anne Plochowietz, Abhishek Mazumder, Achillefs N. Kapanidis

**Affiliations:** 1 Biological Physics Research Group, Clarendon Laboratory, Department of Physics, University of Oxford, Oxford, United Kingdom

## Abstract

Single-molecule fluorescence has been highly instrumental in elucidating interactions and dynamics of biological molecules in the past two decades. Single-molecule fluorescence experiments usually rely on one of two detection geometries, either confocal point-detection or wide-field area detection, typically in a total internal reflection fluorescence (TIRF) format. However, each of these techniques suffers from fundamental drawbacks that limit their application. In this work, we present a new technique, solution wide-field imaging (SWiFi) of diffusing molecules, as an alternative to the existing methods. SWiFi is a simple extension to existing objective-type TIRF microscopes that allows wide-field observations of fast-diffusing molecules down to single fluorophores without the need of tethering the molecules to the surface. We demonstrate that SWiFi enables high-throughput ratiometric measurements with several thousands of individual data points per minute on double-stranded DNA standard (dsDNA) samples containing Förster resonance energy transfer pairs. We further display the capabilities of SWiFi by reporting on mobility and ratiometric characterization of fluorescent nanodiamonds, DNA Holliday junctions, and protein-DNA interactions. The ability of SWiFi for high-throughput, ratiometric measurements of fast-diffusing species renders it a valuable tool for the single-molecule research community by bridging between confocal and TIRF detection geometries in a simple and efficient way.

## Significance

This work establishes a new type of single-molecule method that involves the ratiometric tracking of freely diffusing single molecules in wide-field illumination. The method helps bridge the capabilities of the existing detection geometries of confocal observation and surface-immobilized wide-field imaging by concurrently removing the constraint of surface tethering while still allowing for high throughput and longer observation times than the confocal geometry. Furthermore, the method gathers mobility data that are absent in the methods mentioned above and adds another layer of information to be used to monitor interactions and identify different species.

## Introduction

In the past two decades, single-molecule fluorescence techniques have been highly instrumental in exploring biological mechanisms and interactions both in vitro and in vivo (1, 2, 3, 4).

Single-molecule fluorescence is implemented on two popular detection geometries: point-detection geometries that involve confocal configuration and area-detection geometries that involve wide-field optics. Confocal single-molecule approaches offer high temporal resolution and can be combined with single-molecule Förster resonance energy transfer (smFRET) to provide kinetic information (5, 6, 7, 8, 9, 10). However, typical solution-based confocal smFRET implementations are inherently limited in throughput and cannot infer conformational dynamics at timescales longer than ∼1 ms; as a result, these approaches cannot address molecular dynamics and reaction kinetics at timescales relevant to most complex biological machinery.

These shortcomings can be overcome by viewing surface-immobilized molecules using total internal reflection fluorescence (TIRF) microscopy, which enables wide-field monitoring of 100–1000 molecules simultaneously and achieves much higher throughput per unit time compared to confocal approaches; the gathered individual time traces allow monitoring of dynamics and individual heterogeneity (11, 12, 13, 14). Nevertheless, the need to tether molecules to a surface limits the type of molecules that can be investigated; further, nonspecific interactions of molecules with the surface require sophisticated surface passivation procedures and can lead to artifacts that complicate data analysis and lower the potential throughput.

As a result, several approaches have been developed for wide-field monitoring of single molecules away from the surface; in all cases, the fluorescent molecules are confined in two or three dimensions. One approach that uses three-dimensional confinement relies on surface tethering of small vesicles (∼100 nm in diameter) that encapsulate the molecules of interest (15,16). This approach resolves many problems related to the direct surface tethering of the molecules while maintaining the advantages of wide-field monitoring. However, it is hard to precisely control the number of molecules within the vesicles, which leads to complex signals and lower throughput; further, the study of molecular interactions and the exchange of small molecules (e.g., nucleotides) with the environment is not straightforward and requires introducing pores on the vesicles (16).

Other wide-field approaches rely on restricting the motion of molecules to a depth similar to or smaller than the depth of field of the microscope. One such approach is convex-lens-induced confinement (17), which confines molecules between an adjustable convex lens and a coverslip and allows for long detection times of molecules in solution while concurrently limiting out-of-focus fluorescence to increase the signal/noise ratio (SNR), thus lowering the required illumination intensity and associated photobleaching. A similar approach uses a fabricated cavity between electrostatically charged surfaces to create an electrostatic potential that can capture single charged molecules for times that can extend to many seconds (18). Other confinement approaches use either polydimethylsiloxane (PDMS) (19) or glass (20) nanofluidics to restrict molecules to the observation plane. Although these approaches have been successful in increasing throughput while offering relatively long observation times for each molecule, they require sophisticated and costly apparatus. Furthermore, because the surfaces are within the field of view (FOV), time-consuming surface treatment is required as well to prevent nonspecific interactions. These requirements have limited the wide adoption of these approaches to date.

Three-dimensional confinement is also naturally occurring inside bacterial cells and was used to track fluorescent proteins (21) and electroporated DNA (22). Such cells are placed flat on glass coverslips and have diameters of ∼600–1000 nm; this geometry brings essentially all intracellular fluorescent molecules into focus.

Here, we introduce a single-molecule method that employs wide-field monitoring of single molecules in solution (solution wide-field imaging, or SWiFi) without any physical confinement; SWiFi enables ratiometric measurements of thousands of individual molecules per minute, together with single-molecule tracking to report on their diffusion and dynamics. We demonstrate the ability of SWiFi to track particles of various sizes, including individual fluorophores that diffuse at high speeds exceeding 100 *μ*m^2^/s. The method reports reliably on the FRET values and corresponding structural signatures of static double-stranded DNA (dsDNA) standards, the dynamic behavior of Holliday junctions, the diffusion and ratiometric intensity properties of fluorescent nanodiamonds (NDs) in solution, and the dissociation kinetics of RNA polymerase bound to a promoter DNA fragment. Our approach is a simple extension to existing TIRF microscopes and can help bridge the gap between confocal and surface-immobilized approaches.

## Materials and Methods

### Preparation of DNA constructs

DNA constructs of FRET standards were purchased from IBA Lifesciences (Göttingen, Germany), as well as labeled HJ and promoter DNA constructs. Nonlabeled DNA of the Holliday junction (HJ) strands were purchased from Sigma-Aldrich (Dorset, UK). The FRET standard constructs were labeled with either Cy3B or ATTO647N as described previously (23). FRET standards and promoter DNA were annealed at 1 *μ*M of each strand in annealing buffer (50 mM Tris-HCl (pH 8), 100 mM NaCl, 5 mM EDTA) on a thermocycler (Mastercycler; Eppendorf, Wesseling-Berzdorf, Germany) and kept at −20°C until use. HJ strands were annealed at 4 *μ*M for the R and H strands and 2 *μ*M for the B and X strands in 10 mM Tris-HCl (pH 8), 50 mM NaCl (24) and kept at −20°C until use.

### DNA sequences

For the sequences in Table 1, “bio”-indicates biotin, X indicates a 5′-C6-Amino-T labeled with ATTO647N, and Y indicates a 5′-Amino-C6 labeled with Cy3B unless stated otherwise.

**Table 1:** Sequences of DNA Constructs Used in this Study

Name	Sequence
T1B18	Y-5′-TAAATCTAAAGTAACATAAGGTAACATAACGTAAGCTCATTCGCG-3′-bio
	5′-ATTTAGATTTCATTGTAXTCCATTGTATTGCATTCGAGTAAGCGC-3′
T1B8	Y-5′-TAAATCTAAAGTAACATAAGGTAACGTAAGCTCATTCGCG -3′-bio
	5′-ATTTAGAXTTCATTGTATTCCATTGTATTGCATTCGAGTAAGCGC-3′
HJ	R: 5′-XCCACCG-CTCTTCTCAACTGGG-3′, X is ATTO647N-C6-Amino-C
	B: 5′-CCCTAGCAAGCCGCTGCTACGG-3′
	H: bio-5′-CCGTAGCAGCGAGAGCGGTGGG-3′
	X: 5′-YCCAGTTGAGAGCTTGATAGGG-3′, Y is Cy3B-C6-Amino-C
lacCons+2(−39, +25)-(+15/ATTO647N)	5′-AGGCTTGACACTTTATGCTTCGGCTCGTATAATGT-GTGGAATTGTGAGAGCGGATAACAATTTC-3′
	5′-XCCGAACTGTGAAATACGAAGCCGAGCATCGGCA- CAACCTTAACACTCTCGCCTATTGTTAAAG-3′

### Sample preparation

FRET standard experiments in all systems were carried out by adding 20 *μ*L of the following imaging buffer to a coverslip: 50 mM Tris-HCl (pH 8), 100 mM NaCl, 1 mM TROLOX, 1% w/v glucose, 1 mg/mL glucose oxidase, 50 *μ*g/mL catalase. DNA concentrations ranging from 8 to 80 pM, and glycerol concentrations between 0 and 80% v/v were added according to the specific experiment and are detailed in the Results.

For surface experiments of HJ, 2 mg/mL biotinylated bovine serum albumin (Sigma-Aldrich) in 50 mM Tris-HCl (pH 8) was incubated on a glass coverslip for 1 min, washed with annealing buffer, and incubated with 0.5 mg/mL neutravidin in 0.5× PBS for 1 min, washed again, and incubated with 1 nM HJ for 1 min before washing with annealing buffer. Both surface and solution experiments were carried out with the following buffer: 10 mM Tris-HCl (pH 8), 50 mM NaCl, 25 mM MgCl_2_, 1 mM Trolox, 1% w/v glucose, 1 mg/mL glucose oxidase, 40 *μ*g/mL catalase, and 76% v/v glycerol.

For ND experiments, a sample of 40 nm NDs (Adámas Nanotechnologies, Raleigh, NC) was sonicated for 10 min. The NDs were diluted 100-fold into a 50% v/v glycerol/water solution before mounting on a coverslip for imaging.

For RNA polymerase (RNAP)-DNA dissociation experiments, 50 nM RNAP holoenzyme (New England Biolabs, Ipswich, MA) and 10 nM promoter DNA (LacCons+2) labeled at +15 position of the template strand with ATTO647N (IBA Lifesciences) were incubated in KG7.3 buffer (40 mM HEPES-NaOH (pH 7.3), 100 mM potassium glutamate, 10 mM MgCl_2_, 1 mM dithiothreitol, 5% v/v glycerol) for 20 min at 37°C. The solution, without heparin challenge, was then diluted to a concentration of 100 pM in terms of DNA concentration into an imaging buffer (KG7 buffer with 1 mM Trolox (6-hydroxy-2,5,7,8-tetramethylchroman-2-carboxylic acid), 1% w/v glucose, 1 mg/mL glucose oxidase, and 40 *μ*g/mL catalase). Salt-dependent dissociation measurements of the RNAP-DNA complex were conducted by adding the relevant amount of NaCl to the imaging buffer and imaging after 2 min on the microscope.

### Instrumentation and imaging conditions

Alternating laser excitation (ALEX) measurements of HJ and FRET standards were performed on a custom-built objective-type TIRF microscope (23). A green (532-nm Cobolt Samba; Cobolt, Solna, Sweden) and a red (635-nm Cube; Coherent, Santa Clara, CA) laser were combined using a dichroic mirror and coupled into a fiber optic cable. The fiber output was focused into the back focal plane of the objective (100× oil immersion, numerical aperture 1.4; Olympus, Tokyo, Japan) and displaced from the optical path such that laser light was incident at the slide-solution interface at an angle either greater than the critical angle for surface experiments or slightly lower than the critical angle for solution experiments. ALEX was implemented by directly modulating the red laser and by an acousto-optic modulator (ISOMET, Manassas, VA) for the green laser. Data were acquired with 50 ms exposure time. Excitation powers of 2.5 mW (green) and 1 mW (red) were used for surface experiments, whereas excitation powers of 5 mW (green) and 2 mW (red) were used for solution experiments. Fluorescence emission was collected by the objective and separated from the excitation light by a dichroic (545/650 nm; Semrock, Rochester, NY) and cleanup filters (545 nm long pass filter; Chroma, Bellows Falls, VT and 633/25 nm notch filter; Semrock). The emission signal was focused on a rectangular slit to crop the image and then spectrally separated using a dichroic (630 nm dichroic long pass mirror, 630DRLP; Omega, Norwalk, CT) into two emission channels. The channels were imaged side by side onto an EMCCD camera (iXon 897; Andor, Belfast, Northern Ireland, UK).

Continuous wave experiments to determine diffusion constants of the different samples, as well as FRET histograms of FRET standards, were conducted on a custom-built compact TIRF microscope (“Nanoimager2”). Green and red (532 and 640 nm, 1 W; CNI, Changchun, China) fiber-coupled lasers were connected to the fiber input of a compact (21 × 21 × 14 cm^3^) microscope body and incident on the back focal plane of the objective (100× oil immersion, numerical aperture 1.4; Olympus) at an angle slightly below the critical angle with powers between 50 and 500 mW. The resulting fluorescence was separated by a dichroic mirror (650 nm longpass; Semrock), and the two resulting channels were imaged side by side on a scientific complementary metal–oxide–semiconductor (CMOS) camera (Orca flash 4 v2; Hamamatsu, Hamamatsu City, Japan). The camera’s chip was binned 2 × 2 to create a 1024 × 1024 pixels image. The acquisition software was custom build using MATLAB (The MathWorks, Natick, MA). Experiments were conducted at 24 ± 1°C as measured on the coverslip in the illuminated area. Exposure times varied between 40 ms for monitoring dsDNA in 80% glycerol and down to 2.5 ms for dsDNA in aqueous solution and Cy3B in 30% glycerol.

Fluorescence correlation spectroscopy (FCS) experiments were conducted on a custom-built confocal setup controlled by a bespoke LabVIEW (National Instruments, Austin, TX) program. A green laser (532 nm; Cobolt Samba) operating at 30–100 *μ*W was used to illuminate a diffraction-limited volume through a 60×, 1.35 NA, UPLSAPO 60XO objective (Olympus). The resulting fluorescence was projected onto an avalanche photo diode and fed to a hardware correlator (Flex02-02D; http://correlator.com). The correlation curves were fitted by nonlinear fitting in PyCorrFit software (25) with the following function, which describes the fluctuations in a three-dimensional elongated Gaussian volume with a non-negligible triplet population:

$$G\left(\tau \right)=\frac{1}{n}\frac{1}{\left(1+\tau /\tau_{diff}\right)}\frac{1}{\sqrt{1+\tau /\left(SP^{2}\times \tau_{diff}\right)}}\left(1+\frac{Te^{-\tau /\tau_{trip}}}{1-T}\right),$$

where *n* represents the effective number of molecules within the illuminated volume, *τ*_*diff*_ is the diffusion time of the molecule within the illuminated volume, SP is a measure of the elongation of the volume in the optic axis, *T* is the fraction of molecules in the triplet state, and *τ*_*trip*_ is the triplet lifetime.

ND experiments were conducted on a NanoImager (Oxford Nanoimaging, Oxford, UK). The NDs were excited at 532 nm at 100 mW of power at an angle of 49° to the optical axis with 50 ms exposure, and their emission was imaged on two spectral channels to extract ratiometric intensity values.

In all experiments, glass slides were sonicated twice in pure water for 10 min and washed with water after each sonication. The slides were left to dry in a laminar hood before use. Experiments were conducted at room temperature of 24°C.

Anisotropy experiments were conducted on a steady state fluorimeter (Photon Technology International, Birmingham, NJ) with *λ*_ex_ = 640 nm and *λ*_em_ = 665 nm. Excitation and emission slits were set to 10 nm. 50 nM RNAP holoenzyme (New England Biolabs) and 5 nM promoter DNA (LacCons+2) labeled at +15 of the template strand with ATTO647N (IBA Lifesciences) were incubated in KG 7.3 buffer (40 mM HEPES-NaOH (pH 7.3), 100 mM potassium glutamate, 10 mM MgCl_2_, 1 mM dithiothreitol, 5% glycerol) for 20 min at 37°C. Fluorescence anisotropy values of the promoter DNA, the RNAP-promoter DNA complex, and the RNAP-promoter DNA complex in presence of increasing concentration of NaCl were measured. For the salt challenge experiments after the addition of NaCl to appropriate concentrations, the solution was incubated for 2 min, after which anisotropy values were stable for the length of the measurement (∼3 min). For the determination of the relatively bound fraction, the anisotropy of DNA alone was considered as unbound while the anisotropy of the complex at 100 mM potassium glutamate was considered to represent anisotropy of a solution in which all DNA molecules are bound by the RNAP. The fraction of bound RNAP after addition of NaCl was then calculated as

$$RBF=\frac{AN_{c}-AN_{DNA}}{AN_{KG7.3}-AN_{DNA}},$$

where *AN*_*c*_ is the anisotropy of the solution under a specific NaCl content, *AN*_*DNA*_ is the DNA alone anisotropy, and *AN*_*KG*7.3_ is the anisotropy of the fully bound DNA to RNAP.

#### Single-molecule tracking analysis

The software was written in MATLAB (The MathWorks). First, the two channels are separated, and each channel is segmented to produce a binary image of individual blobs using the adaptive Otsu Method (26), with a sensitivity chosen by the user to maximize correct segmentation of the molecules. Size exclusion is then applied to eliminate false positives (mostly single or a few pixels), as well as large aggregates (larger than 150 pixels in area). Localization is achieved by an intensity-weighted centroid calculation of the segmented pixels of each blob. A nearest neighbor algorithm eliminates spots that are within 1.5 times the maximum allowed distance (MAD) between molecules to minimize false assignments of localizations to tracks. The localizations are fed to an assignment algorithm adopted from a published method (27), which assigns localization to tracks by minimizing the following cost function: *C* = *Δx*^2^ + *Δy*^2^ + MAD × *Δt*^2^, where *Δx*, *Δy* are the spatial distances between tracks and localizations and *Δt* is the time between frames. Multiplying the MAD with *Δt* ensures the algorithm will always prefer to connect localizations within the maximum distance between subsequent frames instead of connecting localizations between later frames. Distances above MAD are given an effective infinite cost to prevent physically impossible assignments. The minimization of the cost function is then achieved by a commercial solver (Gurobi optimization). After completion of the tracking procedure, the distribution of steps for each axis is calculated, and MAD is determined as three times the standard deviation, which for a Gaussian distribution should account for 99% of the steps. The tracking is then repeated until good agreement is achieved between MAD and the calculated value. Individual tracks’ diffusion coefficients were calculated directly from the MSD using the relation

$$\Delta x^{2}=4D\Delta t,$$

where *Δt* is the frame rate. To calculate the ensemble apparent diffusion coefficient, the mean-square displacement (MSD) curve of each axis separately in the track was defined as

$$p\left(n\right)=\frac{1}{N-n+1}\sum_{k=1}^{N-n+1}{\left(X_{k+n}-X_{k}\right)}^{2},$$

where *N* is the number of steps for a specific track. Assuming ergodicity, the ensemble MSD curve can then be calculated as the mean of the individual curves:

$$P\left(n\right)=\frac{1}{M}\sum_{k=1}^{M}p\left(n\right)=2Dn\Delta t+\sigma^{2}-\frac{4}{6}D\Delta t,$$

where *M* is the number of tracks for which *p*(*n*) exists, *σ* is the localization accuracy, and the last term is the result of motion blur (28). From the equation above, it can be seen that for pure diffusion, the curve is linear but does not intersect the origin because of the contributions of the localization error and motion blur. The ensemble apparent diffusion coefficient was calculated from a fit to the linear slope of the ensemble MSD curve because it is not biased by localization error and motion blur to the same extent as specific points in the curve. The calculation for each axis separately allowed for observing when significant drift in the sample was present, and only experiments in which the linear slopes of both axes coincided were used. A minimum of three points on the curve were used for calculation, in which the number of points was chosen so as to improve the accuracy of the linear fit. Hydrodynamic radii were calculated from the diffusion coefficients based on the Stokes-Einstein equation:

$$D=\frac{k_{B}T}{6\pi \eta R_{h}},$$

where *k*_*B*_ is the Boltzmann constant, *T* is the temperature, and *η* is viscosity of the solution. *η* was determined based on the known water/glycerol ratios and the measured temperature using an empiric formula (29).

For FRET tracking, a “greedy” near-neighbor algorithm was used to connect localization to tracks below the MAD. Intensity values were calculated from the total intensity of the segmented area of each blob after removing the mean local background noise around the blob by taking the mean of a window of two pixels in each direction around the bounding box of the blob. Tracks were produced from localizations in either the green or red channel, irrespectively. The apparent FRET value, *E^∗^*, for each localization was calculated as the ratio between the intensity in the red channel/sum of intensities in both channels. In cases in which no localization was found in a specific channel, the localization in the other channel was used to infer the position from which to calculate the intensity for the absent localization.

For both algorithms, a minimum of two steps is required for a track to be considered for analysis to eliminate false tracks.

For ALEX tracking, a channel for acceptor excitation and emission is added, which has its own set of segmentation parameters. The tracking algorithm is the same as in FRET tracking. Tracks with a minimum of four localizations (i.e., two localizations in donor excitation and two in acceptor excitation) or a larger even number are used to calculate FRET values. Both sequences of DADA and ADAD, where D stands for donor and A for acceptor, were used to increase throughput at the expense of less efficient filtering of donor-only species.

The software can be accessed through GitHub at the following address: https://github.com/barakgilboa/SWiFi-software.

## Results

### Concept of SWiFi

To perform wide-field imaging of single molecules in solution without any physical confinement, we considered a simple illumination geometry that provides a depth of illumination that is higher than TIRF but much smaller than in epi-illumination. We thus employed highly inclined and laminated optical sheet microscopy (30) along with dual-color imaging, which has been used successfully for single-molecule tracking and smFRET studies in living bacteria, allowing measurements of molecular mobility and structure in vivo (31,32).

**Figure 1:**
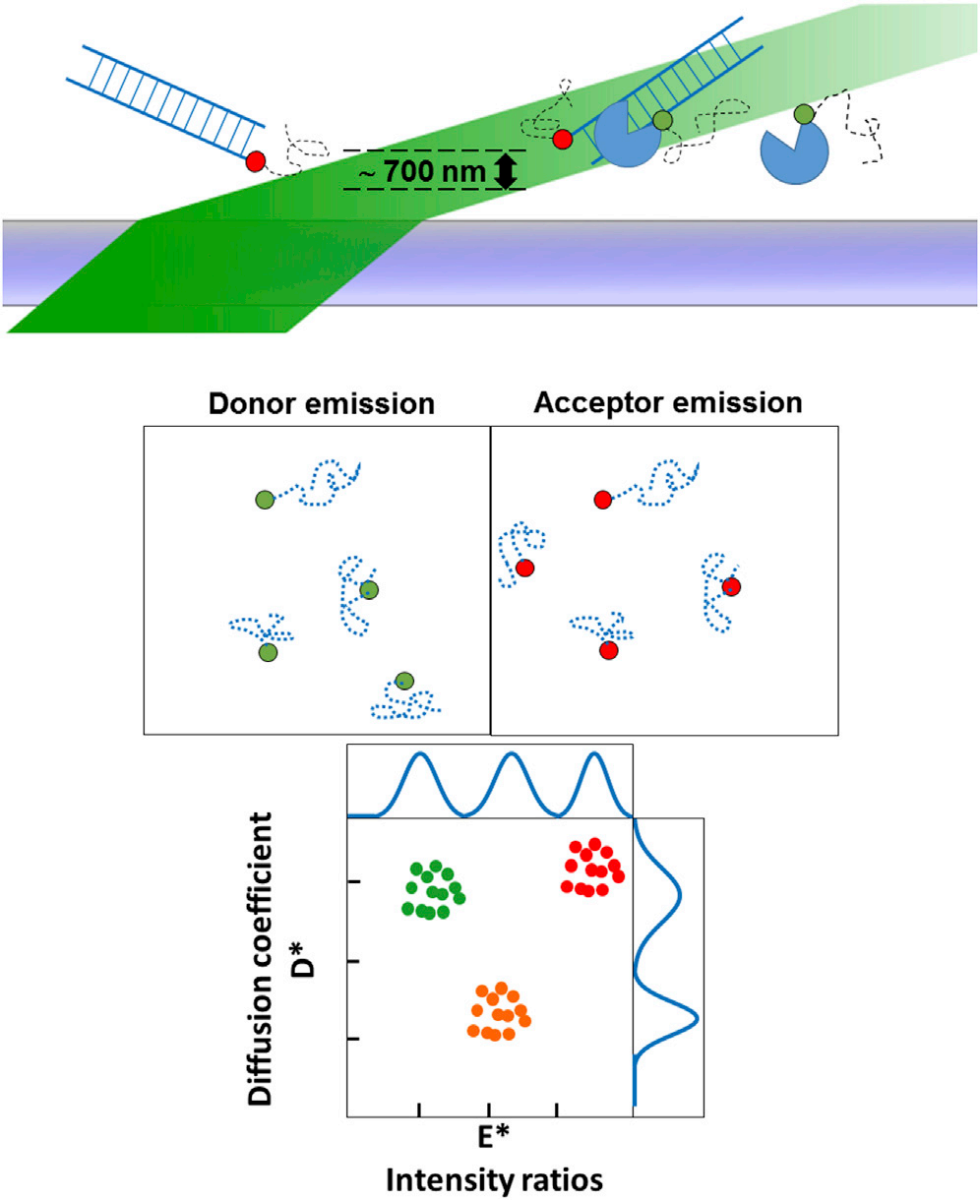
Schematic representation of SWi-Fi. (*Top*) A sketch of the illumination pathway showing the thin sheet created by illumination in an oblique angle. A red-labeled dsDNA and a green-labeled DNA-binding protein diffuse inside the illuminated volume, either alone or as a complex. (*Middle*) Illustration of the three species from the top as observed on two channels in SWiFi. The complex has identical tracks in both channels, whereas the other species are only seen in their respective channels. (*Bottom*) The information gathered from the diffusing molecules is used to build *E^∗^*-*D^∗^* two-dimensional histograms, in which ratiometric or other intensity-related information is correlated to diffusive behavior. For the system depicted at the top, the green-labeled proteins by themselves will give rise to a cluster with low ratiometric value with high diffusion rate; the red-labeled DNAs will give rise to a cluster with high ratiometric value with high diffusion rate, whereas the complexes will give rise to an intermediate ratiometric value with low diffusion rate cluster (*orange*). To see this figure in color, go online.

The angle of illumination was slightly below the critical angle and created a thin light sheet that illuminates the sample at an oblique angle. Within the illuminated volume, the effective observation depth is dominated by the axial point spread function (PSF), which has a full width half maximum of ∼610 nm for a 600-nm emission in our setup (Fig. 1 *A*). We note a slightly higher value because most molecules can be detected even with intensities that are lower than half their maximum intensity. However, the actual observation depth strongly depends on the brightness of the molecule and the diffusion rate, which determines the extent of motion blur. This illumination achieves a compromise between the large illumination depth achieved by epi-illumination and the high SNR achieved by TIRF. We further slow down the diffusion of molecules by increasing viscosity, e.g., using glycerol, to observe fast-diffusing molecules and to increase the overall observation time of individual molecules. The SWiFi approach tracks fluorescent molecules (e.g., dsDNA and DNA-binding protein in Fig. 1 *A*) as they diffuse freely in the FOV. The resulting fluorescence is imaged on two emission channels of a sensitive camera (Fig. 1 *B*), reporting on fluorescence intensities, intensity ratios (or FRET), and molecular mobility (e.g., apparent diffusion coefficients and intensity ratios, Fig. 1 *C*), which enable observation of molecular interactions and associated conformational changes. The combination of mobility data and ratiometric intensities help distinguish the different species in the solution (Fig. 1 *C*).

To maximize the photon count for the diffusing species, we used high excitation intensities (>100 mW), and bright fluorescent probes; notably, any photobleached molecules are continuously replenished because of diffusion, requiring no mechanical motion of the sample or the stage to access nonbleached molecules.

### Determination of diffusion constants for single DNA molecules

Visualizing freely diffusing single molecules in solution has been a challenge for wide-field microscopy owing to the relative low fluorescence signal of single fluorophores, which is lowered further because of the large motion blur of the diffusing molecules. To characterize whether SWiFi can indeed visualize single molecules with sufficient SNR, we examined a 45-bp dsDNA labeled at the 5′ end with FRET donor Cy3B and at position 18 with FRET acceptor ATTO647N (T1B18, with FRET efficiency E_0_ ∼ 0.45 (23)) because it has a well-defined hydrodynamic radius and its intensity should be similar to those expected in ratiometric single-molecule measurements.

**Figure 2:**
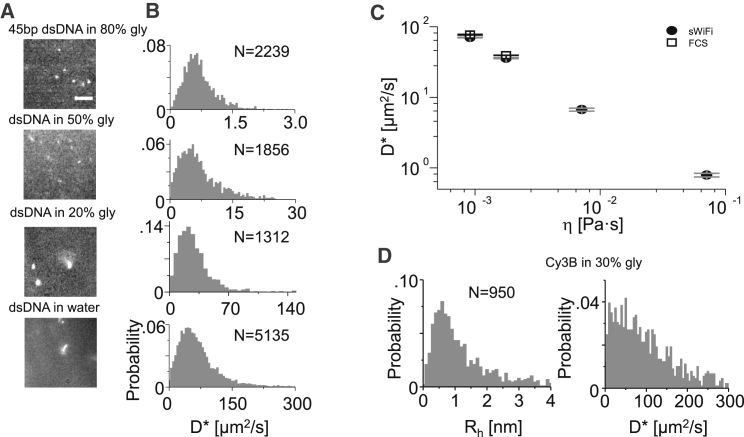
Characterization of DNA standards and Cy3B mobility in glycerol-in-water solutions. (*A*) The image of the T1B18 DNA standard at different glycerol concentrations at 30 ms exposure (80, 50% glycerol, laser power at 250 and 350 mW, respectively) and 20 ms exposure (20, 0% glycerol, power at 500 mW). Scale bars represent 5 *μ*m. (*B*) Diffusion coefficient distributions of the single molecules in the four viscosity regimes. *N* indicates number of individual tracks. (*C*) Average diffusion coefficients in SWiFi and FCS versus viscosity. Circles indicate SWiFi Diffusion coefficients for dsDNA in four different viscosities. Open squares show diffusion coefficients for 0 and 20% glycerol in FCS measurements. Error bars define the standard error of the mean obtained from five separate measurements. (*D*) Hydrodynamic radii distribution (*left*) and diffusion coefficient distribution (*right*) of Cy3B in 30% glycerol (2.5 ms exposure and 500 mW power).

The dsDNA was tracked in solutions of different viscosity, which affected the diffusion of the tracked molecules and led to changes in the images of the molecules in solution (Fig. 2 *A*). In 80% glycerol (*top*, Fig. 2 *A*), the observed image of a molecule is symmetric with a Gaussian implied width of ∼300 nm, similar to the optimal PSF of ∼260 nm that can be obtained on the surface under our imaging conditions (30 ms exposure at 532 nm excitation). At 50% glycerol, the image becomes more elliptical and blurrier with a width of ∼450 nm (*second panel*, Fig. 2 *A*). Finally, in 20% glycerol and in pure water (*bottom panels*, Fig. 2 *A*), each image is unique and cannot usually be approximated by a Gaussian function. Based on these observations, the centroid method of segmented blobs (see Materials and Methods) was chosen over Gaussian fitting for localization because it gives an objective estimation of the position and deals well with both well-defined and blurred images (33).

Tracking the molecules under these conditions allows us to relate the observed images to their underlying diffusion constants (Fig. 2 *B*). The apparent diffusion coefficients D^∗^ (see Materials and Methods for definition) for the DNA construct showed broad distributions with means of 0.8, 6.7, 35.8, and 70 *μ*m^2^/s for the buffer containing 80, 50, 20, and 0% glycerol, respectively; the standard deviations of D^∗^ distributions were 60–100% of the mean D^∗^ values. To test the predictability and repeatability of solution Wi-Fi, we tracked molecules in five separate acquisitions taken from the same experiment for each viscosity regime and calculated the mean diffusion coefficient and the standard error of the mean (Fig. 2 *C*, *top*). The error rates were small and ranged from 1% (for the aqueous solution samples) to 6% (for the 80% glycerol measurement), proving the method’s robustness.

To verify the precision of the calculated diffusion coefficients, we conducted comparable confocal experiments and performed FCS analysis to infer molecular mobility (Fig. 2 *C*, *bottom*). Because the actual shape of the confocal volume was not known, the curves were compared to the results produced by the donor dye of the DNA constructs, Cy3B (with an NHS-ester modification), whose diffusion coefficient in solution was determined by comparison to the well-known FCS standard, rhodamine 6G (R6G). In aqueous solution, the FCS-based Cy3B diffusion time was 19% slower than R6G, indicating a diffusion coefficient of 334 *μ*m^2^/s and a hydrodynamic radius (*R*_*h*_) of ∼0.73 nm, based on the known values for R6G (34) (see Fig. S1). The FCS-determined diffusion time for our 45 bp DNA was ∼4.3 times slower than Cy3B, indicating a diffusion coefficient of 77 *μ*m^2^/s, which is only ∼10% higher than the value found by SWiFi. Because hydrodynamic radii are inversely proportional to diffusion coefficients, we can infer a hydrodynamic radius of ∼3.2 nm for T1B18 based on the value found earlier for Cy3B. This value is in close agreement with a model of short dsDNA diffusion in water (35). The FCS measurements also showed good agreement in 20% glycerol, with diffusion rates of 39.4 and 35.8 *μ*m^2^/s for FCS and SWiFi, respectively. At higher glycerol concentrations, FCS can no longer be trusted to correctly report on diffusion rates for several reasons; first, the addition of glycerol alters the refractive index, which changes the confocal volume. Second, *R*_*h*_ of the Cy3B standard may change in such high glycerol concentrations, as was previously seen for R6G (36), and therefore, it cannot be used as a proxy for the confocal volume. Last, bleaching can become a serious issue at such long dwell times within the spot (85 ms for T1B18 in 80% glycerol), which will lead to a reduced apparent diffusion time and hence an increased diffusion coefficient.

To establish the limit of SWiFi in tracking fast-diffusing molecules, we successfully tracked single Cy3B molecules in 30% glycerol solution, which has ∼3 times the viscosity of water (Fig. 2 *D*; Video S1), and measured a mean diffusion coefficient of 111 *μ*m^2^/s; to our knowledge, this is the fastest value measured by wide-field single-molecule tracking to date. This value corresponds to a hydrodynamic radius of 0.79 nm, in excellent agreement with the value of 0.73 nm obtained using FCS. Diffusion at this rate in an aqueous solution at room temperature will indicate *R*_*h*_ of just 2.2 nm. This size is comparable to the measured *R*_*h*_-value of 2.4 nm for the small (27 kDa) green fluorescent protein (GFP) (37), proving the method is capable of tracking labeled biomolecules of such small size and larger in aqueous solutions.

Video S1. SWiFi Video of Cy3B-NHS Ester in 30% Glycerol Exposure time is 2.5 ms and illumination power is 500 mW. Playback speed is 1/16 of actual acquisition speed. Scale bar is 5 μm.

### Ratiometric tracking of DNA FRET standards

**Figure 3:**
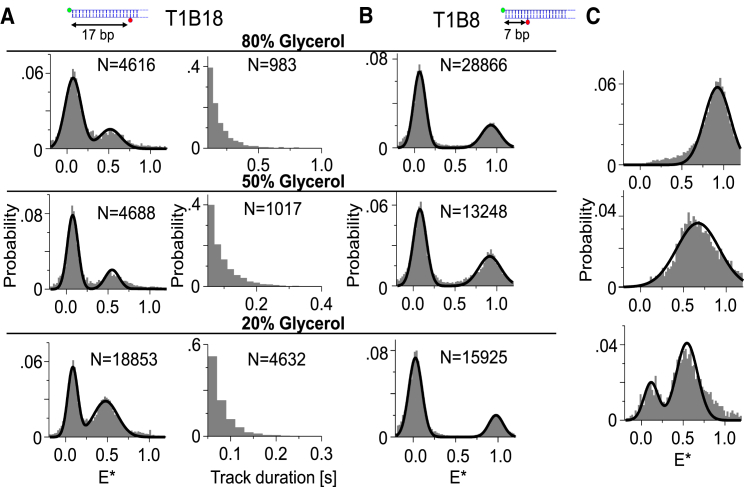
Ratiometric measurements of diffusing DNA standards. (*A*) FRET distributions (*left column*) and track duration distributions (*right column*) of intermediate FRET standard, T1B18, in the three viscosity regimes. (*B*) FRET distributions of high FRET standard, T1B8, in three different viscosity regimes. Exposure times are 50, 30, and 20 ms for the 80, 50, and 20% glycerol solutions. (*C*) FRET distributions of standards after applying methods to remove acceptor bleaching. (*Top*) Tracking in red channel only of T1B8 in 80% glycerol. (*Middle*) Red-channel-only tracking of T1B18 in 80% glycerol. (*Bottom*) ALEX tracking of T1B18 in 50% glycerol. To see this figure in color, go online.

Ratiometric and multicolor measurements are essential components in single-molecule experiments. To characterize our ability to perform ratiometric measurements using SWiFi, we examined two DNA FRET standards: T1B8 (with a 7-bp donor-acceptor separation and E = 0.88) and T1B18 (with a 17-bp donor-acceptor separation and E = 0.45). The strands were imaged and tracked in dual-channel mode in 80, 50, and 20% glycerol. For the high FRET standard, we recovered a bimodal distribution, with a high apparent FRET value of E^∗^ ∼ 0.92 and a low FRET value of E^∗^ ∼ 0.06 (Fig. 3 *A*). These values were calculated from ∼29,000 individual data points collected in 2.5 min from 7013 individual tracks for the 80% glycerol sample; further, ∼12,300 data points were acquired in ∼40 s from ∼3700 individual tracks for the 50% glycerol solution. The low apparent FRET population is common to all FRET experiments and arises from inactive acceptor, due to either imperfect labeling or, more commonly, acceptor bleaching or blinking; this population amounted to ∼66% of the population for both the 80 and 50% glycerol samples. The standard deviation of the measured FRET value was 0.11 in all viscosities, somewhat larger than surface experiments (23) but similar to the value obtained in cells (22).

For the intermediate FRET standard (Fig. 3 *B*), we recovered apparent FRET values of 0.52 for the 80 and 50% glycerol samples and a value of 0.47 in 20% glycerol (Fig. 3 *B*, *left column*). Surprisingly, the low glycerol solution exhibited a lower fraction of acceptor photobleaching (45% versus 68% at the higher concentrations) despite being studied using higher excitation power than the rest (500 mW versus 300 mW entering the objective); this result may be attributed to the differences in triplet population, which can affect the photobleaching rate. Consistent with this interpretation, the triplet population changes dramatically as inferred from FCS (Fig. S1; Supporting Materials and Methods), rising from close to 0% in water to 12–14% in 20 and 50% glycerol and 44% in 80% glycerol. Because most photobleaching occurs from the triplet or other long-lived excited states, a large triplet state population will lead to faster photobleaching.

To establish the observation timescale of individual molecules in SWiFi, we examined the distribution of track durations for the detected molecules. The change in track duration distribution in different diffusion regimes is illustrated with T1B18 (Fig. 3 *A*, *right column*): in 80% glycerol solution (for which a 50-ms exposure time was used), a mean track length of ∼0.2 s was found for 983 individual tracks, which equates to ∼5 frames. Although this number of data points will usually not allow for inferring dynamic rates, there exists within this population a 5% fraction with 10 or more frames, which is already sufficient for analysis on a cumulative basis. At 50% glycerol (with 20-ms exposures), a mean track length of 95 ms was measured, and 10 frames were recorded for ∼5% of the population as well. In 20% glycerol (20-ms exposures), the mean track length was 88 ms, and 10 frames were recorded for ∼2% of the population.

We also considered two approaches to reduce or otherwise handle the presence of acceptor bleaching (and thus the donor-only species) from our measurements because it can become a serious issue when trying to measure low FRET values. In the first one (Fig. 3 *C*, *top* for the 80% glycerol sample of T1B8), tracking is conducted exclusively in the acceptor channel, and the values of the donor channel are then measured based on the acquired positions in the red. This method has proven very effective in removing the acceptor-only species for high-FRET samples but showed bias toward high FRET values when the intermediate FRET standard was examined (*E^∗^* = 0.67, Fig. 3 *C*, *middle*).

Additionally, we used ALEX as an efficient method to resolve different photophysical species in FRET measurements (38). Specifically, we combined ALEX illumination with tracking (see Single-Molecule Tracking Analysis in Materials and Methods), and only tracks that had even numbers of four or higher data points, i.e., two localizations when illuminating with the green laser and two when illuminating with the red laser, were used for analysis. The requirement of signals both in the donor-excitation and acceptor-excitation frames for two frames each at least, significantly reduced the number of tracks with acceptor photobleaching (Fig. 3 *C*, *bottom*, E^∗^ = 0.54, 50% glycerol solution). The remaining donor-only species can be attributed to either coincidence of molecules wrongly associated into tracks because of relatively high concentration for this experiment, as well as the inclusion of tracks, which start in acceptor excitation in which the final donor excitation might occur after acceptor photobleaching (see single molecule tracking analysis in Materials and Methods). Unlike the previous method, ALEX provides objective filtering of the underlying FRET values.

### Observing conformational dynamics in HJs

**Figure 4:**
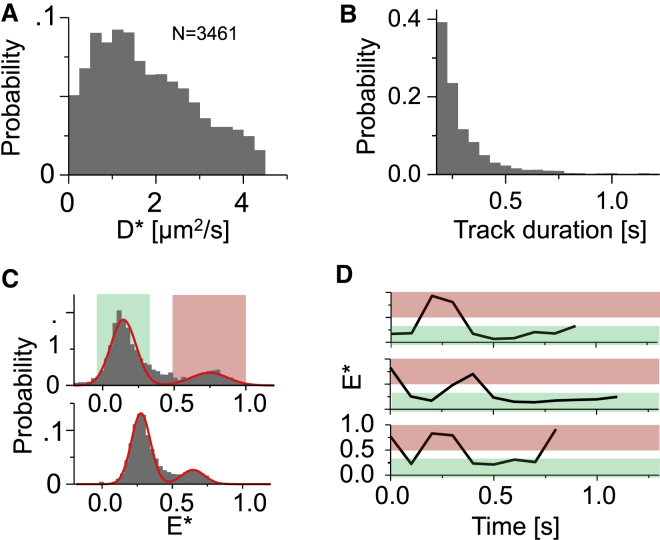
Kinetics and dynamic switching of an HJ. (*A*) Diffusion coefficient distributions of HJ in 76% glycerol. *N* is the number of tracks used in the histogram. (*B*) Track duration distributions of the same sample. (*C*) FRET distributions in solution (*top*) and on the surface (*bottom*) of HJ. The green and red strips indicate a band of two standard deviations from the peak value. (*D*) Individual time traces of single HJs in solution. The red and green strips correspond to the values in (*C*). To see this figure in color, go online.

Elucidating kinetic rates and dynamics of biomolecules are major uses of single-molecule experiments. To demonstrate the ability of SWiFi to probe molecular dynamics and their kinetics, we examined a well-studied model system for conformational dynamics, a DNA HJ (24,39). The junction is comprised of four DNA strands, two of which are labeled at their 5′ end with either Cy3B or ATTO647N. In the presence of Mg^2+^, the HJ we used undergoes large conformational changes that lead to interconversions between two well-separated FRET states (40). The strands were tracked using ALEX tracking because it virtually eliminated donor-only species and, at 76% glycerol, exhibited diffusion rates of ∼2 *μ*m^2^/s (Fig. 4 *A*). In total, 3461 tracks of three frames (0.1 s) or more were obtained over a period of ∼7 min, of which 1981 tracks had four frames or more, as needed for FRET measurements (see Materials and Methods). Track lengths ranged from 0.2 to more than 2.5 s long, with a mean track length of 0.3 s (Fig. 4 *B*). Tracks of 10 frames (0.5 s) or more accounted for 5% of the tracks.

The observed FRET distribution, based on ∼4700 individual observations (Fig. 4 *C*, *top*), showed two distinct peaks at 0.18 and 0.75 apparent FRET efficiency, as compared to 0.27 and 0.65 for equivalent surface experiments (Fig. 4 *C*, *bottom*). The difference in FRET values is likely due to the lower SNR in the SWiFi measurements, which led to underestimation of lower intensities close to the background level, thus pushing FRET values toward the edges of the FRET range. The standard deviations for the two distributions in solution (0.09 and 0.12 for the low and high FRET populations) were similar to those obtained from the equivalent surface results (0.07 and 0.08). The ratio of low/high FRET populations, which reflects the equilibrium constant for the interconversion, was found to be ∼3.7 in solution, very similar to the ∼4.1 obtained for the surface experiments, supporting the method’s ability to quantify dynamic transitions accurately. Furthermore, the equilibrium ratio between the conformers measured by SWiFi was essentially identical to the ratio measured on the surface in a purely aqueous solution, indicating the high glycerol did not alter the energetic landscape of the structure. Both experiments also revealed a third population of intermediate FRET efficiency, which is the result of FRET averaging due to switching of molecules within a single exposure. Although not necessarily required to infer transition rates, dynamic interconversions (see Fig. 4 *D* for some examples) allow distinguishing specific FRET species, as well as using methods such as hidden Markov modeling to correctly identify FRET states and dwell times.

### Characterization of size and photophysical properties of NDs

We also used SWiFi to study the properties of fluorescent NDs, which are promising new nanomaterials for applications such as temperature sensing (41), magnetic sensing (42), and biological imaging (43,44). These applications rely on a nitrogen vacancy (NV) defect that acts as a fluorescent color center and emits a broad emission spectrum with a peak either at 670 nm (for a negatively charged state, NV^−^) or at 610 nm (for the neutrally charged state, NV^0^).

**Figure 5:**
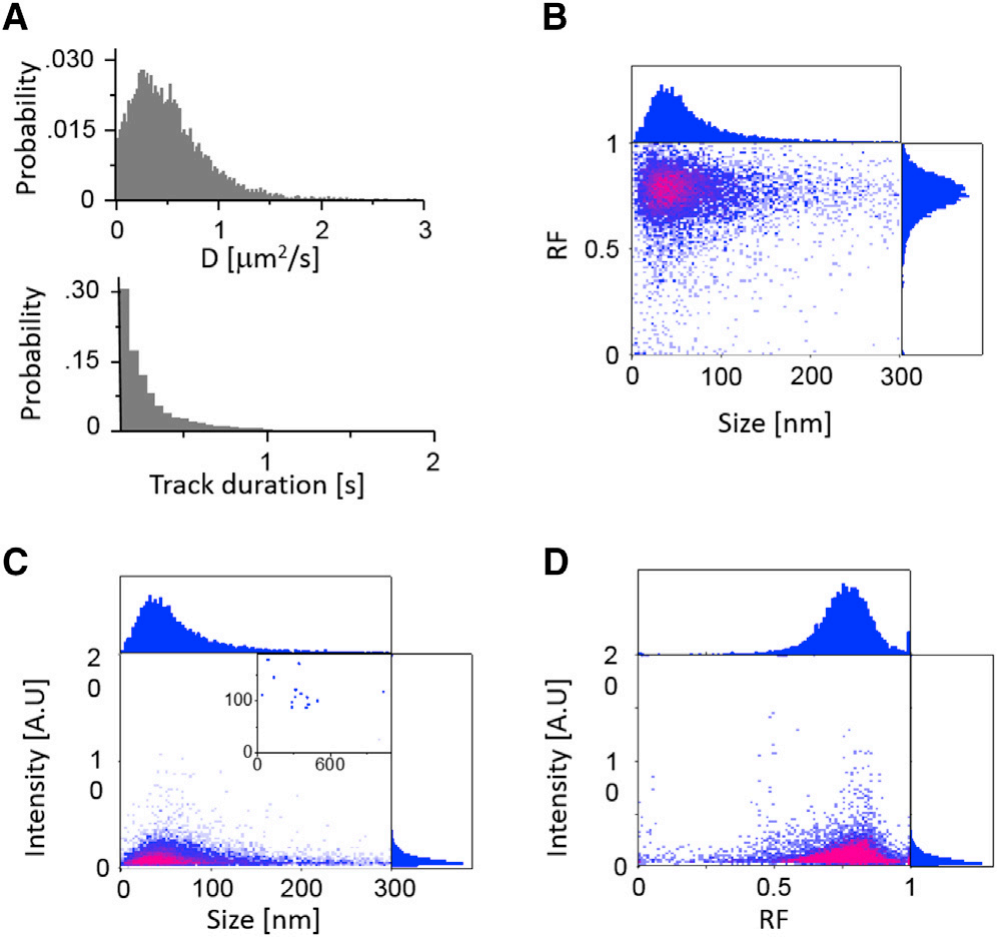
Characterization of ND sample using SWiFi. (*A*) Distribution of diffusion constants (*top*) and track duration (*bottom*) of individual NDs. Experiments were conducted at 50 ms exposure in a 50% v/v glycerol/water solution. (*B*) Bivariate distribution of size versus mean ratiometric value for the individual tracks. (*C*) Bivariate distribution of size versus intensity for the individual tracks. Inset: distribution of a small group of very bright particles. (*D*) Bivariate distribution of mean ratiometric value versus intensity for the individual tracks. To see this figure in color, go online.

Although much work has been done to characterize NV centers in bulk diamonds, the photophysics of NDs is not well-understood yet. The high throughput provided by SWiFi, as well as its size characterization, make it ideal for studying the size distributions and photophysics of NDs populations. The NDs used here were 40 nm in diameter on average, having 1–2 NV centers per particle, with 30% of the population having no NV centers at all. The use of 50% glycerol ensured extended observation time per particle and allowed characterization that is more sensitive. Based on ∼9000 individual tracks, the measured diffusion coefficients were 0.2–0.7 *μ*m^2^/s, with a mean of ∼0.6 *μ*m^2^/s (Fig. 5 *A*, *top*). At this diffusion rate, track lengths extended for up to 3 s with a mean of 375 ms, or 7.5 frames (Fig. 5 *A*, *bottom*). For each track, a diffusion coefficient, and therefore an apparent hydrodynamic radius was established, together with its mean intensity and mean ratiometric value “red fraction” (RF), which was defined equivalently to FRET, i.e., the ratio between intensity of emission in the red channel (R)/the total intensity in both channels (R + G). These parameters were then used to create two-dimensional histograms of RF, intensity, and *R*_*h*_ for the NDs (Fig. 5, *B*–*D*).

The distribution of *R*_*h*_ (Fig. 5 *B*, one-dimensional projection on *top*) was centered on 40 nm, with a median of 60 nm and a mean of 130 nm, which indicates aggregation of the supplied 40-nm ND sample. RF was centered at 0.75 (Fig. 5 *B*, one-dimensional projection on the *right*), as expected for NV^−^ charge state in our setup, and exhibited a Gaussian distribution. The intensity-size distribution (Fig. 5 *C*) did not show any clear correlation between the size and the measured intensity, which can be attributed to the low number of NV centers per particle. A very small subgroup of larger particles (or aggregates) exhibited enhanced brightness (*inset* of Fig. 5 *C*).

The RF-intensity distribution (Fig. 5 *D*), on the other hand, exhibited two clear species, centered at ∼0.76 and 0.5 RF-values. The 0.5 group most likely arose from emission of the NV^0^ state and was also observed with the same sample in surface experiments (45). The 0.5 RF species exhibited higher intensity on average, with a small subgroup of less than 10 particles exhibiting values two orders of magnitude higher than the median intensity and corresponding to sizes of >300 nm (part of the particles depicted in the *inset* of Fig. 5 *C*); the 0.5 RF species also amounted to just 2% of the total population, and observing it is a testament to the method’s capabilities of detecting small subpopulations in heterogeneous samples. An additional species with RF close to 0 originated from contaminants within the solution, which appeared somewhat in the green channels of every solution measured, or alternatively, due to surface contaminants on the ND itself, which could be distinguished from the NV center fluorescence because of their fast bleaching. A closer observation reveals a smaller group in between the two species, which may indicate some interconversion between the states. Such behavior was seen before in bulk diamonds and more recently in NDs on a coverslip surface (45, 46, 47).

### Determining the salt dependence of RNA polymerase-promoter complex

**Figure 6:**
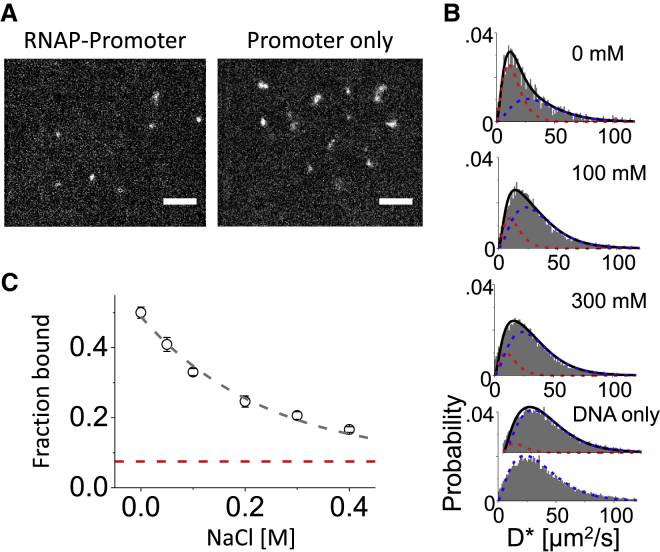
Salt dependence of RNAP interaction with a promoter DNA. (*A*) Images of promoter DNA labeled with ATTO647N after incubation with RNAP holoenzyme (*left*) and by itself (*right*) are given. Scale bars, 5 *μ*m, exposure time 5 ms. (*B*) Diffusion coefficient histograms of RNAP-DNA complex at differing NaCl concentration. The black line is the fitting of two species, the red is the contribution of the complex, and the blue is the free promoter DNA. At the bottom is the single fit (*lowest*) and double fit (*higher*) of the promoter DNA alone. (*C*) The fraction of bound DNA as a function of NaCl concentration. Error bars indicate 95% confidence intervals of the double fitting. Dashed gray line is an exponential fit to the data. Dashed red line marks the fraction of the supposed bound molecules when a double fit is applied to the DNA alone (∼0.07). To see this figure in color, go online.

DNA-protein interactions are important in many biological processes and have been studied extensively at the single-molecule level. SWiFi can contribute to study of such interactions by reporting on FRET values as described above, as well as by reporting on change in mobility of the molecules as a complex is formed or dissociates. To illustrate the latter ability, we studied the dissociation of a complex of RNAP with a 65-bp-long fluorescently labeled promoter DNA (LacCons+2; see Materials and Methods). After incubation of RNAP with the promoter DNA (see Materials and Methods), a large reduction in diffusion of the DNA is seen, both visually (as the individual images become more localized and less blurred; Fig. 6 *A*; Videos S2 and S3) and in the distribution of diffusion coefficients (Fig. 6 *B*, *top* and *bottom*). To determine the fraction of DNA bound to RNAP, the promoter DNA was first imaged alone, and its diffusion coefficient was measured.

Video S2. SWiFi Video of Promoter DNA Alone Exposure time is 5 ms and illumination power is 350 mW. Playback speed is 1/8 of actual speed. Scale bar is 5 μm.

Video S3. SWiFi Video of Promoter DNA after Incubation with RNAP Holoenzyme Exposure time is 5 ms and illumination power is 350 mW. Playback speed is 1/8 of actual speed. Scale bar is 5 μm.

The probability distribution of diffusion coefficients was fitted to a function of the form (32)

(1) $$f_{D}\left(x\right)=\frac{1}{2}{\left(\frac{3}{D}\right)}^{3}x^{2}e^{-\frac{3x}{D}}.$$

The resulting fit suggests a diffusion coefficient of *D* ∼ 39 *μ*m^2^/s for the free promoter DNA, which correlates to *R*_*h*_ ∼5 nm (Fig. 6 *B*, *bottom*). We then used this result to infer the diffusion coefficient of the complex (Fig. 6 *B*, *top*) as incubated in KG7.3 buffer (see Materials and Methods). The diffusion coefficients distribution of the complex was fitted with two species, one being the free promoter DNA and the other being the RNAP-DNA complex:

(2) $$f_{D}\left(x\right)=\frac{27}{2}\left(\frac{a}{D_{1}^{3}}x^{2}e^{-\frac{3x}{D_{1}}}+\frac{\left(1-a\right)}{D_{2}^{3}}x^{2}e^{-\frac{3x}{D_{2}}}\right),$$

where *a* is the fraction of bound molecules, *D*_1_ is their diffusion, and *D*_2_ = 39 *μ*m^2^/s is the diffusion coefficient of the free DNA. The fitting (Fig. 6 *B*, *top*) suggested the solution consisted of 49% RNAP-DNA complexes diffusing at ∼15.6 *μ*m^2^/s (R_h_ ∼13.4 nm) and 51% the free promoter DNA. After finding the two diffusion coefficients, the stability of the complex was challenged by addition of increasing amounts of NaCl to the solution after initial incubation (see Materials and Methods). For 50 mM NaCl, our measurements suggested that the sample re-equilibrates within 2 min of observation time (Fig. S3), reaching a metastable state that eventually dissociates completely within an hour. For concentrations above 50 mM NaCl, equilibrium was reached by the time the first acquisition was over (20–30 s from the addition of the salt), and no discernible additional dissociation was observed until the completion of the acquisition (50–60 s in total). For concentrations above 50 mM NaCl, equilibrium was reached by the time the first acquisition was over (20–30 s from the addition of the salt), and no discernible additional dissociation was observed until the completion of the acquisition (50–60 s in total). Therefore, all the experiments were conducted after 2 min in the regime in which the bound fraction was stable within the acquisition time. The molecules were imaged and tracked, and the fraction of bound molecules was found based on fitting the two populations of the free and bound promoter (Fig. 6 *B*, *middle panels*). The results illustrate an exponential dependence with a coefficient of 0.24 M^−1^ in the fraction of bound molecules with the increase in NaCl concentration (Fig. 6 *C*), toward the value achieved when fitting the free promoter with the two species (Fig. 6 *B*, *bottom* and *dashed red line*, Fig. 6 *C*). This behavior was similar to the behavior observed in anisotropy experiments with the same constituents (Fig. S4; Materials and Methods), although no direct comparison can be made because of the 50-fold difference in the concentrations of RNAP and DNA in the final solutions.

## Discussion

We demonstrated sensitive, high-throughput, and multimodal detection of single molecules and particles in solution without any physical confinement. Our method is fully compatible with molecular species of different size, structure, and chemistry. The SWiFi method represents a simple yet powerful extension of the capabilities of conventional objective-type single-molecule microscopes (using TIRF or epi-illumination fluorescence) to observations of freely diffusing molecules. SWiFi is a straightforward extension to existing TIRF microscopes and does not require any additional apparatus. The method can monitor both mobility and fluorescence ratios concurrently and is able to record dynamic conformational changes.

SWiFi can track molecules as small as 27 kDa in aqueous solutions and provides ratiometric intensity measurements of molecules diffusing at rates similar to those of small proteins in the cell cytoplasm. For mobility studies, SWiFi offers several advantages over FCS. First, individual diffusion rates are calculated directly from observations without the need of a model to which to fit the results and thus does not suffer from errors arising from fitting FCS data with an inadequate model. Ensemble diffusion coefficients are calculated from a simple linear fit to the MSD curve, which is again far simpler than the equivalent FCS models. Accurate FCS measurements require detailed knowledge of the confocal volume, which is a function of the laser wavelength, numerical aperture of the objective, the distance above the surface, the refractive index of the sample, and the coverslip thickness. Because it is rarely possible to precisely know all of the above factors, the use of standard dyes whose diffusion rates are known is common to infer diffusion rates. However, even for R6G in aqueous solutions, there exist a wide variety of reports regarding its diffusion rates in FCS (48), partly because of the use of less than pure samples that include additional fluorescent species that cannot be directly detected in the correlation curve (48). Unlike FCS, SWiFi is a single-molecule technique, which allows it to differentiate between subpopulations in solution in a manner similar to work based on single-molecule tracking in cells (32,49).

Confocal smFRET is limited in throughput because of the need of avoiding coincidence of molecules within the confocal volume. This limitation leads to low data acquisition rates of up to 200 molecules/min. Surface-tethered wide-field imaging can achieve up to 100 molecules per FOV with an EMCCD camera and up to ∼1000 molecules per FOV on an sCMOS camera. SWiFi enables a high data throughput for ratiometric measurements, probing more than a thousand molecules per minute and gathering 5000–15,000 FRET data points per minute; this is similar to the throughput achieved by using a complex multifocus confocal setup with a single photon detector array (50,51).

Observation times also differ widely between the methods. Both confocal and SWiFi are limited by their observation volume, which leads confocal to have observation times on the order of 1 ms and SWiFi to have a range between a few milliseconds for a short dsDNA in aqueous solution to a few tens of milliseconds in low viscosity, and up to a few hundred milliseconds in high viscosity. Larger complexes can be observed for up to a few seconds under similar conditions. Surface-tethered imaging, which is limited only by bleaching, can typically achieve ∼10–100 s of observation time per molecule.

The capabilities of the method do not come without cost. Observing fast-diffusing molecules requires short exposure times and therefore high intensity, which leads to faster photobleaching. Fast molecules allow only a short observation time before leaving the thin sheet of illuminated volume, and their motion blur lowers the SNR of the image and thus increases the noise in ratiometric measurements. As a result, ratiometric measurements required the slowing down of molecules, which we achieved by increasing the solution viscosity with the addition of glycerol. Nevertheless, we successfully measured FRET standards in 20% v/v glycerol, which has a viscosity just twice that of pure water; the measured diffusion constant of 28.5 *μ*m^2^/s was slightly higher than the diffusion constant measured for free GFP in the cytoplasm of eukaryotic cells (52) and three times that of free GFP in bacteria (4). Indeed, the same DNA construct that was used here with slight changes (singly labeled and without biotin) was electroporated and tracked before in bacterial cells (22). For two such constructs with different labels, apparent mean diffusion coefficients were found to be 0.76 and 0.92 *μ*m^2^/s. These apparent diffusion coefficients translate to real diffusion coefficients that are two to three times larger (21). Such diffusion coefficients will be observed under our experimental conditions when using ∼70% v/v glycerol solution, which infers an effective viscosity that is 30-fold that of water. Although the slow diffusion observed in cells can in part be explained by nonspecific interactions with the cell constituents, most of the slowdown in diffusion relative to aqueous solutions is due to the crowded environment within the cells. Therefore, ratiometric measurements using SWiFi can be performed for small molecules in viscosity environments that resemble the cell cytoplasm. Extended observation times for single molecules can also be achieved by attaching molecules to a large body such as a liposome (53). Alternatively, the use of an inert polymer matrix can differentially alter the effective viscosity of the solution depending on the size of the diffusing species (54), which will enable easier distinction between those species. The polymer matrix can also mimic macromolecular crowding in cells (55,56) and does not significantly alter the diffusion of oxygen scavengers and triplet quenchers, which should reduce the triplet population and lower photobleaching.

Methods to slow down diffusion in SWiFi also increase throughput for several reasons: by reducing motion blur, which in turn increases the number of molecules that are being detected; by extending the mean track duration, which in turn increases the amount of usable tracks; and by allowing the use of higher concentrations because of lower frame-to-frame motion, which reduces the minimal near-neighbor distance between molecules required to avoid wrong assignment of molecules to tracks.

Several extensions are available to enhance the capabilities of SWiFi; e.g., stroboscopic illumination can be used to observe fast-diffusing molecules and to capture fast dynamics (57). By exciting the molecules with a strong pulse, which is much shorter than the exposure time, one can achieve significantly less motion blur, higher SNR, and better ability to capture the kinetics of fast switching molecules by avoiding averaging, as was seen in our HJ measurements. Fast scanning of either the stage or the illumination angle at a rate that is much faster than the frame rate can increase the effective illumination volume and thus allow tracking more molecules, as well as extending observation times. When combined with ALEX, SWiFi can also differentiate between different photophysical species in solution and remove a substantial fraction of donor-only contributions.

The additional mobility data give SWiFi a unique advantage compared to its confocal and TIRF counterparts. Because mobility can report on the association of two biomolecules of interest, as we demonstrated, additional information can be gathered from complementary methods such as FRET that can report on conformational changes related to this association, for example.

A specific area in which SWiFi can find wide application is the study of large complexes. These complexes, such as transcription-translation complex (58) and transcription-replication conflicts (59) in bacteria, as well as ion channels (60), can be very challenging to immobilize on the surface in a functional form, whereas their slow diffusion means imaging them in a confocal geometry will require very low concentrations and therefore lower throughput. On the other hand, in SWiFi, the slow diffusion of such large complexes will enable longer exposure times and therefore lower excitation power, less photobleaching, and longer observation times. The slow diffusion will also allow for using much higher concentrations than confocal, thus monitoring many molecules concurrently without the worry of misassigning the molecules to their comparable tracks.

## Author Contributions

B.G., A.N.K., A.M., and M.S. designed the experiments. B.G., A.M., and M.S. performed the experiments. B.G. wrote the tracking software of SWiFi and analyzed the data. B.J. designed, constructed, and wrote the software of the custom TIRF microscope used for most of the experiments. T.J.C., A.P., and B.J. designed and conducted preliminary experiments. B.G. and A.N.K. wrote the article. All authors reviewed the article.

## Supporting Material

Document S1. Supporting Materials and Methods and Figs. S1–S4

Document S2. Article plus Supporting Material
