# Supplementary material for: Confinement-Free Wide-Field Ratiometric Tracking of Single Fluorescent Molecules
Source: Biophys J. 2019 Oct 31;117(11):2141–53. doi: 10.1016/j.bpj.2019.10.033 (PMC6895709; doi:10.1016/j.bpj.2019.10.033)
Supplement: Document S1. Supporting Materials and Methods and Figs. S1–S4 [file mmc1.pdf]

Biophysical Journal, Volume *117*

Supplemental Information

Confinement-Free Wide-Field Ratiometric Tracking of Single Fluorescent Molecules

Barak Gilboa, Bo Jing, Tao J. Cui, Maabur Sow, Anne Plochowietz, Abhishek Mazumder, and Achillefs N. Kapanidis

Confinement-free wide-field ratiometric tracking of single fluorescent molecules

Barak Gilboa[1], Bo Jing[1], Tao J. Cui[1], Maabur Sow[1], Anne Plochowietz[1], Abhishek Mazumder[1], and Achillefs N. Kapanidis[1]

[1]Biological Physics Research Group, Clarendon Laboratory, Department of Physics, University of Oxford, Oxford OX1 3PU, UK

Supplementary information

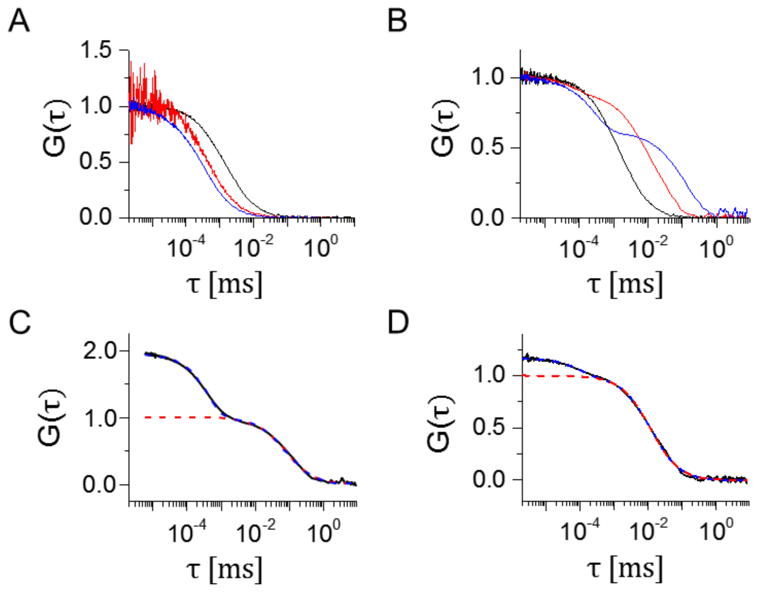

Supplementary figure 1. FCS curves of diffusing molecules. A. FCS curves of Rhodamine 6G (blue), Cy3B (red) and T1B18 (black) in an aqueous solution. B. T1B18 in water (black), 50% glycerol (red) and 80% glycerol (blue). C. T1B18 in 80% glycerol (black) fitted with a model that includes triplet contribution (dashed blue), and with the triplet contribution removed (dashed red). D. T1B18 in 50% glycerol. Markings are identical to C.

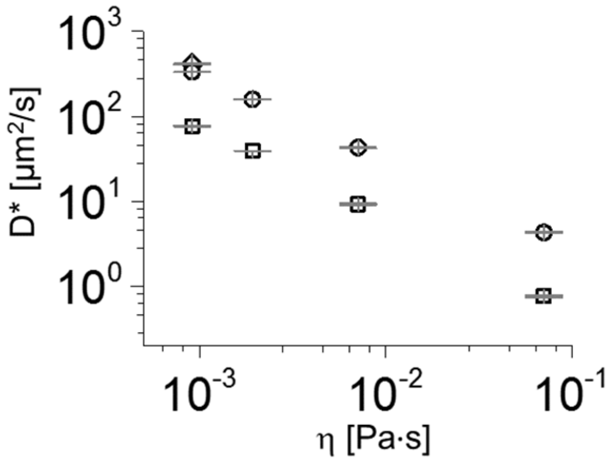

Supplementary figure 2. FCS implied diffusion of Rhodamine 6G (open diamond), Cy3B (open circles) and T1B18 (open squares) in 0%, 20%, 50% and 80% glycerol. Error bars indicate standard error of the mean.

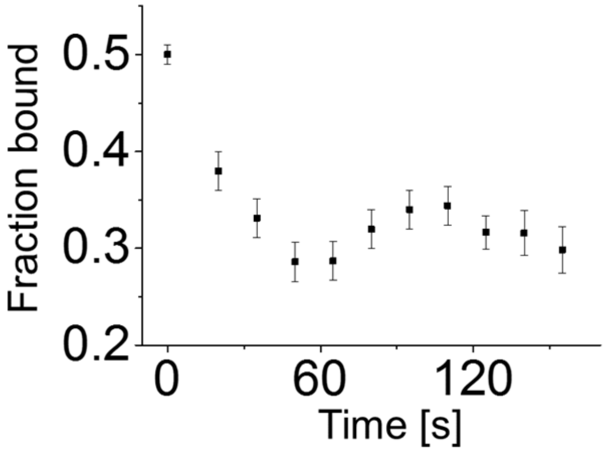

Supplementary figure 3. Fraction of bound DNA to RNAP after addition of 50 mM NaCl vs time. Error bars indicate 95% confidence levels of two species fitting to diffusion coefficients distributions.

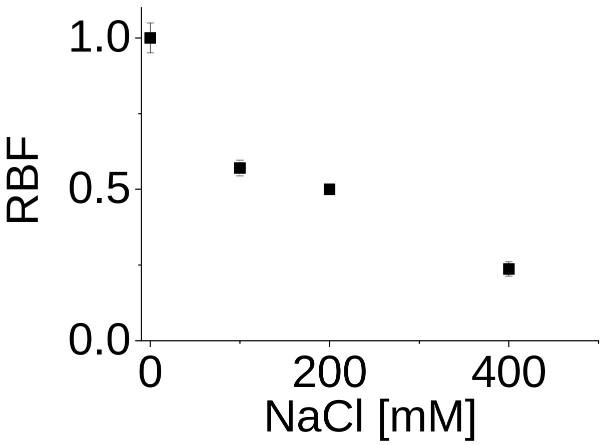

Supplementary figure 4. The ratio of bound fraction (RBF) of RNAP to DNA as a function of added NaCl as inferred from anisotropy measurements.

<u>Captions of supplementary movies</u>

Supplementary movie S1. SWiFi movie of Cy3B-NHS ester in 30% glycerol. Exposure time is 2.5 ms and illumination power is 500 mW. Playback speed is 1/16 of actual acquisition speed. Scale bar is 5 μm.

Supplementary movie S2. SWiFi movie of promoter DNA alone. Exposure time is 5 ms and illumination power is 350 mW. Playback speed is 1/8 of actual speed. Scale bar is 5 μm.

Supplementary movie S3. SWiFi movie of promoter DNA after incubation with RNAP holoenzyme. Exposure time is 5 ms and illumination power is 350 mW. Playback speed is 1/8 of actual speed. Scale bar is 5 μm.

Triplet population in FCS analysis

Molecules were analysed in four different glycerol concentrations, namely 0%, 20%, 50% and 80%. Their FCS curves were fitted to a model of diffusion within a 3D elongated Gaussian. This model proved sufficient for all the molecules in aqueous solution, but in higher glycerol concentrations deviations from the model appeared for short correlation times (sup. Fig 1C-D). To account for these features, a model that also includes triplet lifetime and fraction of the population was used. The model adequately accounted for the changes in the curves, indicating a significant triplet population in high glycerol concentrations. While negligible triplet population is found in water, in 20% and 50% glycerol, Cy3B and T1B18 had ~9% and 12-14% triplet population fraction respectively. In 80% glycerol, Cy3B had ~23% triplet population fraction, and T1B18 had more than 46% triplet population fraction. The high triplet population leads to both lower observed intensity and faster photobleaching of the molecules.
